# Antagonist-mediated down-regulation of toll-like receptors increases the prevalence of human papillomavirus infection in systemic lupus erythematosus

**DOI:** 10.1186/ar3803

**Published:** 2012-04-18

**Authors:** Shui-Lian Yu, Paul KS Chan, Chun-Kwok Wong, Cheuk-Chun Szeto, Suzanne C Ho, Karine So, May MY Yu, So-Fan Yim, Tak-Hong Cheung, Martin CS Wong, Jo LK Cheung, Apple CM Yeung, Edmund K Li, Lai-Shan Tam

**Affiliations:** 1 Department of Medicine and Therapeutics, Prince of Wales Hospital, The Chinese University of Hong Kong, 30-32 Ngan Shing Street, Shatin, New Territories, Hong Kong; 2 Department of Microbioloby, Prince of Wales Hospital, The Chinese University of Hong Kong, 30-32 Ngan Shing Street, Shatin, New Territories, Hong Kong; 3 The Stanley Ho Centre for Emerging Infectious Diseases, Prince of Wales Hospital, The Chinese University of Hong Kong, 30-32 Ngan Shing Street, Shatin, New Territories, Hong Kong; 4 Department of Chemical Pathology, Prince of Wales Hospital, The Chinese University of Hong Kong, 30-32 Ngan Shing Street, Shatin, New Territories, Hong Kong; 5 Centre of Research and Promotion of Women's Health, Prince of Wales Hospital, The Chinese University of Hong Kong, 30-32 Ngan Shing Street, Shatin, New Territories, Hong Kong; 6 Department of Anatomical and Cellular Pathology, Prince of Wales Hospital, The Chinese University of Hong Kong, 30-32 Ngan Shing Street, Shatin, New Territories, Hong Kong; 7 Department of Obstetrics and Gynaecology, Prince of Wales Hospital, The Chinese University of Hong Kong, 30-32 Ngan Shing Street, Shatin, New Territories, Hong Kong; 8 School of Public Health and Primary Care, Prince of Wales Hospital, The Chinese University of Hong Kong, 30-32 Ngan Shing Street, Shatin, New Territories, Hong Kong

## Abstract

**Introduction:**

Prevalence of an abnormal Papanicolaou smear was significantly increased in lupus patients in cross-sectional studies, associated with a higher prevalence of high-risk human papillomavirus (HPV) infection. The nucleic acid-specific Toll-like receptors (TLRs) locate at the endolysosomal compartments and trigger the induction of cytokines for the innate immune response. This study evaluated whether abnormal host innate immune response in lupus patients may enhance HPV persistence.

**Methods:**

Protein levels of TLRs 3, 7, 8 and 9 in cervical epithelial cells of lupus patients and controls with or without HPV infection were assessed using flow cytometry. Characteristics associated with the differential expression of TLRs in systemic lupus erythematosus (SLE) were elucidated. The effect and interferon-stimulated genes (ISGs) (ISG15 and Mx-1) gene expressions were then measured in oncogenic HeLa (HPV18), CaSki (HPV) and C33A (HPV negative) cell lines using flow cytometry and quantitative real-time PCR. *Ex vivo *productions of cytokines and interferon-gamma (IFN-γ) upon TLR ligands stimulations were subsequently measured using cytometric bead array and ELISA.

**Results:**

For subjects with HPV infection, levels of TLR3 and TLR7 were significantly lower in lupus patients compared with controls. Significantly decreased TLRs 7, 8 and 9 levels were observed in HPV-negative SLE compared to healthy controls. For SLE with and without HPV infection, TLR7 and 9 levels were significantly lower in infected SLE than those in HPV-negative patients. Independent explanatory variables associated with down-regulation of TLR7 level included HPV infection and a higher cumulative dose of prednisolone; while a higher cumulative dose of hydroxychloroquine and HPV infection were associated with down-regulation of TLR9 level. In cervical cell lines, TLRs 3, 7, 8, 9 protein levels and antiviral ISG15 and Mx-1 gene expressions were inhibited in two oncogenic HPV types. Functional data showed that the induction of pro-inflammatory cytokines by TLR ligands (R837, ssRNA and ODN2395) was greatly impaired in CaSki and HeLa than C33A cells.

**Conclusions:**

In conclusion, prednisolone and TLR antagonist (hydroxychloroquine) may down-regulate protein levels of TLR7 and TLR9 in lupus patients, thereby decreasing the innate immune response against HPV infection. Upon infection, HPV further down-regulate TLR7 and 9 levels for viral persistence. Furthermore, reduction of nucleic acid-sensing TLRs 7, 8 and 9 in carcinogenic HPVs ensures that the expression of inducible pro-inflammatory cytokines is minimized to prevent the expression of antiviral ISGs (ISG15 and Mx-1) on a biologically relevant antiviral response.

## Introduction

Persistent infection with an oncogenic human papillomavirus (HPV)16 or 18 is thought to be necessary for the development of invasive cervical cancer, particularly among immunocompromised patients [1]. Previous studies have revealed an increase in the prevalence of abnormal Papanicolaou (Pap) smears among patients with systemic lupus erythematosus (SLE) [2,3]. Independent risk factors associated with the development of squamous intraepithelial lesion (SIL) in lupus patients included persistent oncogenic HPV infection and the use of cyclophosphamide [4]. Although the majority of the otherwise healthy individuals clear HPV infection with time, almost half (48.5%) of the newly acquired HPV infections persisted for at least six months in lupus patients [5]. However, none of the clinical, lifestyle, gynecological and treatment regime characteristics was predictive of persistent HPV infection in lupus patients [4].

Innate immune recognition of viral infection triggers antiviral immune responses [6,7]. Viral nucleic acids act as pathogen-associated molecular patterns and are recognized by multiple TLRs. Intracellular TLRs 3, 7, 8 and 9 are involved in the recognition of viral nucleotides, such as double-stranded RNA (dsRNA) (TLR3), single-stranded RNA (ssRNA) (TLR7-TLR8) and DNA (TLR9) [8]. Papillomavirus is a small dsDNA virus. It has been suggested that DNA viruses might produce RNA transcripts that engage TLR3 [9]. Pattern recognition receptor (PRR) signalling can be suppressed by the inhibition of downstream signalling or sequestration of typical viral nucleic acids, thereby inhibiting viral recognition [10]. In SLE patients, factors regulating the levels of TLRs include inflammation, as reflected by systemic lupus erythematosus disease activity index (SLEDAI), which may up-regulate TLR levels in peripheral blood mononuclear cells (PBMCs) [11,12]. In addition, TLR9 can induce anti-dsDNA antibody production, and pro-inflammatory cytokines may also up-regulate TLR levels [13]. On the other hand, factors which may down-regulate TLRs include the use of high-dose prednisolone and TLR antagonists, such as hydroxychloroquine [14,15]. However, whether the abnormal host innate immune response in lupus patients may play a role in enhancing HPV persistence remained unknown.

Type I interferons (IFNs) are key effector molecules of the innate immune system and are essential for the antiviral response towards a plethora of viruses [16]. Nucleic acid-sensing TLRs play a critical role in antiviral immunity by inducing the downstream of IFNs, including IFN-α, IFN-β and IFN-γ, and other TLR-induced inflammatory cytokines [6]. Recent genome-wide transcriptome analysis indicates that the expression of interferon-stimulated genes (ISGs) and pathogen receptors are inhibited by oncogenic HPV types [17-19], but the details of the mechanisms remained uncertain. As many viruses have adopted mechanisms to escape from TLR recognition by blocking IFN-mediated responses induced via TLR-dependent and -independent cascades [17,20,21], carcinogenic HPVs may inhibit both antiviral ISGs and nucleic acid-sensing TLRs for a biologically relevant antiviral response in HPV16, 18 and 31 [17-19,22,23]. Whether these nucleic acid-sensing TLRs, IFN-γ and ISGs (ISG15 and Mx1 (myxovirus resistance)), which have been shown to function as antiviral effectors during HPV infection [17,24] and markers for patients with severe lupus [25], may be different in cell lines harboring HPV16 (CaSki) and 18 (HeLa) genomes compared to HPV-negative (C33A) cells has never been explored.

The aims of this cross-sectional study included the following: 1) To compare the protein levels of TLRs 3, 7, 8 and 9 in cervical epithelial cells of SLE patients and controls with or without HPV infection; 2) To ascertain the potential explanatory clinical and laboratory variables associated with TLR protein levels in cervical epithelial cells; 3) To investigate whether the reduced TLR protein levels has functional consequences for cytokine induction. Our findings showed that prednisolone and hydroxychloroquine may down-regulate levels of TLR7 and TLR9, respectively. Interestingly, upon infection, HPV further down-regulates TLR7 and TLR9 levels in lupus patients, which is consistent with previous reports showing that oncogenic HPV16 may suppress the host immune response by down-regulating the TLR9 transcript and, subsequently, limit its ability to induce the transcription of pro-inflammatory genes for antiviral immune responses [23].

## Materials and methods

### Subjects

One hundred and fifty consecutive female patients attending the lupus clinic at The Prince of Wales Hospital of The Chinese University of Hong Kong, who fulfilled the 1997 American College of Rheumatology revised criteria for the classification of SLE [26], and participated in a prospective study on SLE and cervical intraepithelial neoplasia, were recruited for this cross-sectional study. Details of the previous study have been described elsewhere [4]. Briefly, subjects were eligible for the study if they were married or sexually active. Excluded were subjects who were pregnant or under treatment for invasive cervical cancer. None of the subjects had received HPV vaccine. Twenty-nine age-matched female Chinese volunteers were recruited as controls.

Subjects were divided into four groups based on the following criteria: Group 1 - SLE patients with positive HPV DNA (*n *= 15); Group 2 - SLE with negative HPV DNA (*n *= 31); Group 3 - control subjects with positive HPV DNA (control subjects referred for colposcopy because of abnormal cytology) (*n *= 7); and Group 4 - healthy controls with negative HPV DNA (healthy women for screening at the Well Women Clinic, Hong Kong) (*n *= 22)

All women with abnormal cytology (atypical squamous cells of undetermined significance (ASCUS) or worse) or positive HPV DNA were referred for colposcopy and cervical biopsy to obtain histological diagnosis. Ethics approval has been obtained from the Ethics Committee of The Chinese University of Hong Kong, and informed consent was obtained from all participants according to the Declaration of Helsinki.

### Clinical and laboratory parameters

The study visit included a structured interview with a standardized questionnaire. Clinical assessment and blood collection for the determination of disease activity, gynecologic examination for the collection of specimens for a Pap test, and HPV testing were performed. Medication history for the past 15 years was retrieved from case notes. Physical examinations and laboratory investigations, including complete blood count, liver and renal functions, anti-dsDNA titer, serum complements C3 and C4 levels, were performed at study visit. The SLEDAI and Systemic Lupus International Collaborating Clinics/American College of Rheumatology damage index (SLICC) were evaluated during the clinical assessment [27,28]. Major organ involvement was present if the disease affected any of the following systems: neuropsychiatric, kidney, heart and hematologic (hemolytic anemia, platelet < 100,000/μl). Immunosuppressive agents included prednisolone, hydroxychlorlquine, azathioprine, cyclophosphamide (oral or IV), cyclosporin A (CsA) and mycophenolate mofetil (MMF).

### HPV sampling procedure and identification

The method of cervical sample collection, detection and identification of HPVs has been described previously [29]. Briefly, two cervical samples were collected with a Cervex brush (Rovers Medical Devices, Netherlands) from each woman. The first sample for routine liquid based cytologic examination and the second sample for FACS analysis were immersed in 10 mL of phosphate buffered saline (PBS, Gibco Laboratories, Grand Island, NY, USA) and Dulbecco's modified Eagle's minimal essential medium/F12 medium (DMEM) (Invitrogen, Carlsbad, CA, USA) supplemented with 10% defined fetal bovine serum (FBS) (Invitrogen), respectively. Samples were then agitated vigorously for dislodging the residual cervical cells. After cytologic examination, the remained sample was used for HPV DNA detection. DNA was extracted and then detected by the PGMY polymerase chain reaction (PCR) targeting the consensus region of the HPV L1 gene [29-31]. Samples tested positive for HPV DNA were subjected to Linear Array HPV Genotyping Test that can detect 37 types of HPV. HPV types were grouped as high-risk and low-risk [29]. The second cervical sample collected in 10% FCS-DMEM was immediately transported for flow cytometric analysis.

### Flow cytometric gating for cervical epithelial population

Cervical cell pellets (1 × 10^6 ^cells) were resuspended and blocked with 2% human pooled serum for blocking the non-specific Fc receptors. Leukocytes were phenotyped by staining with anti-CD45 antibody using R-phycoerythin (PE) mouse IgG1κ isotypic control (Biolegend Inc, San Diego, CA, USA) as background control. The cells were then fixed and permeabilized using Fix/Perm solution (BD Biosciences Corp., San Diego, CA, USA). Purified mouse anti-cytokeratin antibody (Millipore Inc., Billerica, MA, USA) and mouse IgG1κ isptypic control (BD Biosciences) were used for intracellular staining, together with a fluorescein iso-thiocyanate (FITC) secondary antibody (Zymed Laboratories Inc., South San Francisco, CA, USA). The side scatter (SSC), forward scatter (FSC) and florescence signal of 10,000 epithelial cells were then acquired and analysed by flow cytometer using CellQuest™ software (FACSCalibur, BD Biosciences).

### Flow cytometric analysis of intracellular TLRs

Serum-blocked cervical epithelial cells, HeLa, CaSki and C33A cells were fixed and permeabilized as previously mentioned. FITC-conjugated antibodies for TLRs 3, 8, 9, PE-conjugated TLR7 antibody (Imgenex, San Diego, CA, USA) and corresponding FITC-conjugated IgG isotypic control (BD Biosciences) and PE-conjugated mouse IgG1, κ (Biolegend) were incubated with cells for 30 minutes at 4°C. The levels of TLRs in 10,000 epithelial cells were assessed using flow cytometer and the results were expressed as mean fluorescence intensity (MFI).

### Cell culture

HeLa (ATCC, CCL-2) and C33A cells (ATCC, HTB-31) were purchased from American Type Culture Collection (ATCC, Manassas, VA, USA) and maintained in MEM-Earle medium (Sigma-Aldrich Corp., St. Louis, MO, USA), supplemented with 10% FBS, 60 mg/l gentamycine and 1% non-essential amino acid (Biochrom, Berlin, Germany). Sodium pyruvate (5%) (Sigma) was added for C33A cells. CaSki cells (ATCC, CRL-1550) obtained from ATCC was grown in RPMI1640 medium (Sigma) containing 10% FBS, 60 mg/ml gentamycine and 1% non-essential amino acid. Cells (5 × 10^5 ^cells) were incubated with or without TLR ligand Poly (I:C) (TLR3 ligand, Sigma) and ODN2395 (TLR9 ligand, InvivoGen, San Diego, CA, USA) at 10 μg/ml each and R837/Imiquimod (TLR7 ligand) and ssRNA (TLR8 ligand) at 5 μg/ml each for 24 h at 37°C in a 5% CO_2 _atmosphere. The cell-free supernatant was harvested and stored at -70°C for subsequent assays of IFN-γ and cytokines.

### Quantitative real-time PCR of ISGs (ISG15 and Mx1) gene expressions

Total RNAs of Caski, HeLa or C33A were extracted using RNeasy Mini Kit (Qiagen Inc., Valencia, CA, USA). All RNA samples were treated with DNase I (Invitrogen) and reversely transcribed to cDNA with TaqMan Reverse Transcription Reagents (Applied Biosystems Inc., Foster city, CA, USA). The mRNA ISG15, Mx1 and GAPDH (endogenous control) was quantified by real-time PCR using SYBR Green probe (Roche Diagnostics Corp., Indianapolis, IN, USA) with the use of Applied Biosystems' 48-well StepOne™ Real Time PCR System. The primers of human ISG15, Mx1 and GAPDH were as follows [32]: ISG15 forward (5'-GGCGGGCAACGAATTCCAGGTGT-3') and reverse (5'-CTCCCCGCAGGCGCAGATTCA-3'); Mx1 forward (5'-TGCTGCATCCCACCCTCTATTAC

T-3') and reverse (5'-GGCGATGGCATTCTGGGCTTTAT-3'); GAPDH forward (5'-ATGGGGAAGGTGAA

GGTCG-3') and reverse (5'-GGGGTCATTGATGGCAACAATA-3'). Real-time PCR was performed in a 25ul reaction mixture containing primers, FastStart Universal SYBR Green master (ROX) reagent (Roche) and cDNA sample in duplicate. The real-time PCR reaction was performed as previously described [33].

### Quantitative analysis of IL-1β, IL-6, IL-8, IL-10, IL-12, TNF-α and IFN-γ

The concentration of cytokines, including interleukin (IL-6), IL-8, IL-10 and IL-12p70, tumor necrosis (TNF)-α and IL-1β in culture supernatant with equal cell number loading, were measured simultaneously using human inflammatory cytokine Cytometric Bead Array (CBA, BD Pharmingen, San Diego, CA, USA) with flow cytometry (FACSCalibur, BD Biosciences) [34]. Human IFN-γ in culture supernatant was assayed by enzyme-linked immunosorbent assay (ELISA) reagent kit (R&D systems, Minneapolis, MN, USA). The range of detection was 15.6 to 1,000 pg/ml for ELISAs and 20 to 5,000 pg/ml for CBA.

### Statistical analysis

Results were expressed as mean ± S.D. for normally distributed data. Non-normally distributed data were expressed as median (interquartile range, IQR). Chi-squared tests were used for categorical variables. For continuous variables, the Student's *t *test or Mann-Whitney U test was used where appropriate. Association between the potential explanatory variables and TLRs level were tested using Chi-squared tests for categorical variables and Pearson's or Spearman's correlation for continuous variables with normal and skewed distribution, respectively. Variables with *P *< 0.05 in the univariate analysis were entered into linear regression analysis (enter). Variables that were skewed were logarithmically transformed before entering the regression analysis. All hypotheses were two-tailed, and *P-*value < 0.05 were considered significant. The Statistics Package for Social Sciences (SPSS for Windows, version 13.0, 2006, SPSS Inc., Chicago, IL, USA) was used for the analysis.

## Results

### Pap smear findings, socio-demographic, behavioral and clinical characteristics

The prevalence of cytological abnormality was significantly higher in HPV-positive subjects (Group 1 vs Group 2: 53% vs 3% and Group 3 vs Group 4: 71% vs 14%) (Table 1). Socio-demographic and gynaecologic characteristics of controls with and without HPV infections (Groups 3 and 4) were similar, except the unemployment rate and the prevalence of having multiple sexual partners were increased in Group 3. In those subjects without HPV infection, a higher prevalence of menopause and unemployment was observed in the SLE patients (Group 2) compared with healthy controls (Group 4). SLE patients with HPV infection (Group 1) had a significantly longer disease duration, a higher prevalence of at least two major organ involvement, hematologic involvement, and the use of hydroxychloroquine when compare to Group 2 (all *P <*0.05) (Table 2). The prevalence of multiple (≥ 2) HPV infections, infection by high-risk or low-risk HPV type, or HPV subtypes was similar in SLE patients (Group 1) and controls (Group 3) (data not shown).

**Table 1:** Pap smear findings, socio-demographic and behavioral characteristics of lupus patients and control subjects ^#^

	SLE patients		Control subjects	
	Group 1 (HPV-positive) (*n *= 15)	Group 2 (HPV-negative) (*n *= 31)	Group 3 (HPV-positive) (*n *= 7)	Group 4 (HPV-negative) (*n *= 22)
Pap smear findings				
Cytological abnormality *	8 (53)	1 (3) ^†††^	5 (71)	3 (14) ^‡‡^
				
Demographic & social characteristics				
Age at study, mean ± S.D. (range), year	45 ± 10 (28 to 58)	47 ± 10 (30 to 63)	41 ± 10 (31 to 59)	44 ± 11 (25 to 62)
Menopause	7 (47)	17 (55)	2 (29)	4 (18) ^§§^
Married	9 (60)	24 (77)	7 (100)	17 (77)
Low education level (high school or lower)	12 (80)	25 (81)	7 (100)	15 (68)
Monthly family income ≤ HK$ 10,000	6 (40)	9 (29)	1 (14)	6 (27)
Alcohol	0 (0)	3 (10)	1 (14)	1 (5)
Current smoker	0 (0)	3 (10)	1 (14)	1 (5)
Employment	7 (47)	11 (35)	2 (29)	18 (82) ^‡‡, §§^
				
Reproductive and gynecologic characteristics				
First intercourse at young age (≤ 17 years)	2 (13)	4 (13)	1 (14)	2 (9)
Multiple sexual partners (≥ 3)	3 (20)	2 (6)	4 (57)	1 (5) ^‡‡^
History of sexually transmitted disease	1 (7)	0 (0)	0 (0)	0 (0)
Oral contraceptives	2 (13)	3 (10)	1 (14)	2 (9)
Condom use by male partner	6 (40)	11 (35)	3 (43)	11 (50)
Current vaginal discharge	0 (0)	2 (6)	0 (0)	1 (5)
Bleeding after intercourse	0 (0)	2 (6)	0 (0)	2 (9)

**^# ^**Values are the number (percentage) unless otherwise indicated. * Refers to atypical squamous cells of undetermined significance or squamous intraepithelial lesions. HPV = human papillomavirus; SLE = systemic lupus erythematosus; Group 1 = SLE with positive HPV DNA; Group 2 = SLE with negative HPV DNA; Group 3 = Control subjects with positive HPV DNA; Group 4 = Healthy controls with negative HPV DNA; ^††† ^*P <*0.001, comparing between SLE patients with and without HPV infection. ^‡‡ ^*P <*0.01, comparing between control subjects with and without HPV infection. **^§§ ^***P *< 0.01, comparing between SLE and control subjects without HPV infection

**Table 2:** Clinical characteristics of patients with systemic lupus erythematosus #

	Group 1 (HPV-positive) (SLE patients = 15)	Group 2 (HPV-negative) (SLE patients = 31)
Clinical features		
SLE duration, mean ± S.D.(range), year	18 ± 8 (6 to 36)	13 ± 6 (5 to 28) ^†^
SLICC, median (IQR)	1 (0 to 2)	0 (0 to 1)
SLEDAI score, median (IQR)	2 (0 to 6)	2 (0 to 4)
Serological features		
Serum complement C3, median (IQR), g/l	0.9 (0.7 to 1.1)	0.8 (0.6 to 1.0)
Serum complement C4, median (IQR), g/l	0.2 (0.1 to 0.3)	0.2 (0.1 to 0.3)
Elevated anti-dsDNA (> 100 IU/ml)	6 (40)	13 (42)
Anti-dsDNA titer, median (IQR), IU/ml	308 (210 to 591)	331 (93 to 572)
Major organ involvement ever		
0	0 (0)	3 (10)
1	1 (7)	9 (29)
≥ 2	14 (93)	19 (61) ^†^
Neuropsychiatric	3 (20)	10 (32)
Nephritis	12 (80)	17 (55)
Serositis	6 (40)	7 (23)
Hematologic	15 (100)	24 (77) ^†^
Immunosuppressive therapy ever	15 (100)	29 (94)
Prednisolone	14 (93)	24 (77)
Hydroxychloroquine	12 (80)	15 (48) ^†^
Azathioprine	9 (60)	16 (52)
Cyclophosphamide (oral or IV)	2 (13)	5 (16)
Cyclosporin A	2 (13)	7 (23)
Mycophenolate mofetil	3 (20)	5 (16)

**^# ^**Values are the number (percentage) unless otherwise indicated. * Refers to atypical squamous cells of undetermined significance or squamous intraepithelial lesions. Anti-dsDNA, anti-double stranded DNA;

IQR, interquartile range; SLE, systemic lupus erythematosus; SLEDAI, systemic lupus erythematosus disease activity index; SLICC, systemic lupus international collaborating clinics/Americal College of Rheumatology damage index. Group 1, SLE with positive HPV DNA; Group 2, SLE with negative HPV DNA; ^† ^*P <*0.05 comparing between SLE patients with and without HPV infection.

### Protein level of TLRs 3, 7, 8 and 9 in the cervical epithelial cells of lupus patients and controls

**Figure 1:**
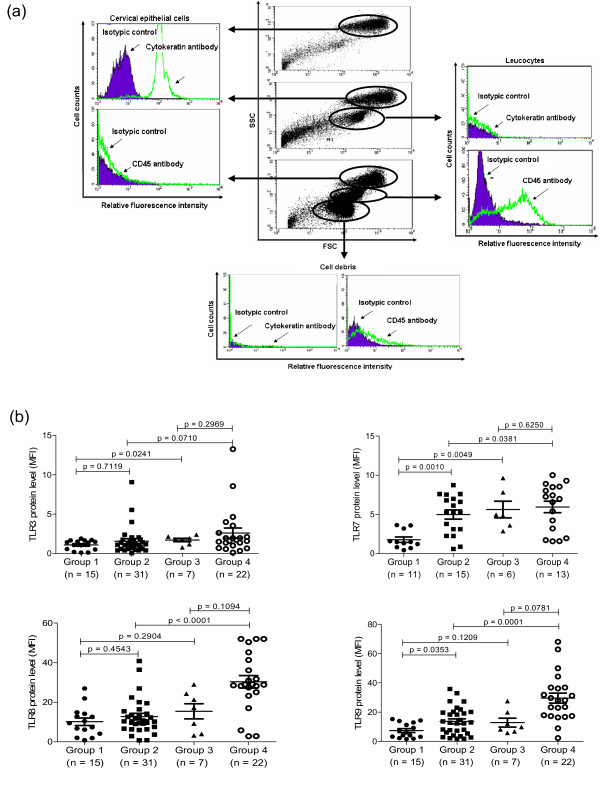
**Protein levels of TLRs 3, 7, 8 and 9 in human cervical epithelial cells**. **(a) **Representative do plot analysis showing the respective gating for cervical epithelial cells (cytokeratin^+ ^CD45^-^), leucocytes (cytokeratin^-^CD45^+^) and cell debris (cytokeratin^-^CD45^-^) based on their forward/sideward scatter characteristics (FSC/SSC) in three independent human cervical samples using flow cytometry. **(b) **The differential levels of TLRs 3, 7, 8 and 9 in gated cervical epithelial cells were shown in scatter plots as mean fluorescence intensity (MFI). Group 1 = SLE with positive HPV DNA; Group 2 = SLE with negative HPV DNA; Group 3 = Control subjects with positive HPV DNA; Group 4 = Healthy controls with negative HPV DNA.

The protein levels of TLRs 3, 7, 8 and 9 in gated cytokeratin^+^CD45^- ^cervical epithelial cells (Figure 1a) of lupus patients and controls were shown in the scatter plots as MFI (Figure 1b). For subjects with HPV infection (Groups 1 and 3), TLR3 and TLR7 levels were significantly lower in lupus patients compared with controls. Significantly decreased levels of TLRs 7, 8 and 9 were observed in HPV-negative SLE compared to healthy controls (Groups 2 and 4). TLR7 and TLR9 levels were significantly lower in HPV-DNA positive than HPV-DNA negative SLE patients (Groups 1 and 2). No significant differences in the levels of TLRs 3, 7, 8 and 9 was found between controls with and without HPV infection (Groups 3 and 4) (all *P *> 0.05).

### Potential explanatory variables associated with TLR levels in lupus patients

Tables 3 and 4 summarized the association between clinical, treatment regime characteristics and levels of TLRs 3, 7, 8 and 9 assessed by univariate analysis (Table 3: continuous variables, Table 4: categorical variables). There was a significantly positive correlation between the SLEDAI and TLR9 level (Table 3). Significantly positive correlation was found between TLR8 level and titers of anti-dsDNA (*P *< 0.05) (Table 3). Regarding the role of immunosuppressants, a higher cumulative dose of prednisolone was inversely correlated with TLR7 level (Table 3). Moreover, TLR9 level was negatively correlated with the cumulative dose of hydroxychloroquine and the duration of azathioprine treatment during the past 15 years. In contrast, TLR3 level was positively correlated with the cumulative dose of CsA (Table 3). The TLR7 level was significantly lower in patients treated with hydroxychloroquine compared to those having never been treated with hydroxychloroquine. Significantly decreased level of TLR7 was found in patients infected with high-risk HPV types compared with those without high-risk HPVinfection (all *P *< 0.05) (Table 4).

**Table 3:** Effects of clinical variables on the TLR levels in lupus patients (continuous variable)

	TLR3			TLR7			TLR8			TLR9		
	n	r	*P*	n	r	*P*	n	r	*P*	n	r	*P*
Clinical characteristic												
SLE duration, year	46	-0.263	0.279	26	-0.187	0.214	46	0.183	0.222	46	0.064	0.672
SLEDAI score	46	-0.004	0.977	26	-0.030	0.885	46	0.161	0.284	46	0.446	0.002 ^††^
Anti-dsDNA titer (> 100 IU/ml)	19	0.272	0.261	13	-0.291	0.334	19	0.498	0.030 ^†^	19	-0.094	0.702
Immunosuppressive therapy												
Prednisolone												
Current dose, mg	36	-0.082	0.636	22	-0.164	0.466	36	0.116	0.502	36	-0.032	0.855
Cumulative dose, gm	38	-0.020	0.898	22	-0.436	0.042 ^†^	38	0.102	0.542	38	0.018	0.916
Duration, months	38	-0.150	0.368	22	-0.223	0.318	38	-0.100	0.555	38	0.017	0.920
Hydroxychloroquine												
Current dose, mg	16	0.261	0.329	10	-0.044	0.905	16	-0.226	0.400	16	0.052	0.848
Cumulative dose, gm	27	-0.073	0.716	15	-0.132	0.639	27	-0.193	0.335	27	-0.399	0.039 ^†^
Duration, months	27	-0.106	0.598	15	-0.179	0.524	27	-0.139	0.488	22	-0.081	0.689
Azathioprine												
Current dose, mg	8	-0.385	0.346	5	-0.671	0.215	8	0.462	0.249	8	-0.089	0.833
Cumulative dose, gm	25	-0.166	0.429	16	-0.012	0.966	25	-0.336	0.101	25	-0.339	0.098
Duration, months	25	-0.036	0.865	16	-0.118	0.664	25	-0.396	0.505	25	-0.440	0.028 ^†^
Cyclophosphamide (oral or IV)												
Current dose, mg	4	-0.316	0.684	2	N.A.	N.A.	4	-0.316	0.684	4	-0.316	0.684
Cumulative dose, gm	7	0.107	0.840	3	N.A.	N.A.	7	-0.107	0.840	7	-0.500	0.267
Duration, months	7	0.571	0.200	3	N.A.	N.A.	7	0.143	0.783	7	-0.393	0.396
Cyclosporin A												
Current dose, mg	-	-	-	-	-	-	-	-	-	-	-	-
Cumulative dose, gm	9	0.783	0.013 ^†^	5	0.800	0.133	9	-0.333	0.948	9	-0.283	0.463
Duration, months	9	0.200	0.613	5	0.900	0.083	9	0.167	0.678	9	0.333	0.385
Mycophenolate mofetil, gm												
Current dose, mg	5	0.359	0.553	3	N.A.	N.A.	5	0.718	0.872	5	0.564	0.322
Cumulative dose, gm	8	0.071	0.935	5	-0.600	0.350	8	-0.429	0.299	8	-0.381	0.360
Duration, months	8	-0.048	0.935	5	-0.700	0.233	8	-0.548	0.171	8	-0.429	0.299

Anti-dsDNA, Anti-double stranded DNA; SLE, systemic lupus erythematosus; SLEDAI, systemic lupus erythematosus disease activity index; TLR, toll-like receptor; **^† ^***P <*0.05, **^†† ^***P <*0.01.

**Table 4:** Effects of clinical variables on the TLR levels in lupus patients (categorical variable)

	TLR3				TLR7				TLR8				TLR9			
	Yes		No		Yes		No		Yes		No		Yes		No	
	n		n		n		n		n		n		n		n	
Clinical manifestation																
Neuropsychiatric	13	1.3 ± 1.0	33	1.5 ± 1.7	8	3.8 ± 2.8	18	3.9 ± 2.7	13	12 ± 7	33	12 ± 9	13	12 ± 7	33	12 ± 9
Nephritis	29	1.2 ± 0.7	17	1.8 ± 2.3	17	3.5 ± 2.5	9	4.5 ± 3.0	29	12 ± 9	17	12 ± 9	29	11 ± 8	17	13 ± 9
Serositis	13	1.3 ± 1.0	33	1.5 ± 1.7	10	3.8 ± 2.3	16	3.9 ± 2.9	13	11 ± 7	33	12 ± 10	13	11 ± 9	33	12 ± 9
Hematologic	39	1.4 ± 1.6	7	1.4 ± 1.3	22	3.2 ± 2.4	4	5.1 ± 1.7	39	13 ± 9	7	8 ± 4	39	11 ± 8	7	18 ± 10
																
High-risk HPV infection	11	1.1 ± 0.6	35	1.5 ± 1.7	8	1.9 ± 1.3	18	4.7 ± 2.7 ^†^	11	10 ± 9	35	13 ± 9	11	8 ± 5	35	13 ± 9
																
Immunosuppressive therapy ever																
Prednisolone																
Ever	38	1.2 ± 1.0	8	2.4 ± 3.0	22	3.5 ± 2.5	4	5.6 ± 3.0	38	12 ± 8	8	13 ± 10	38	11 ± 9	8	16 ± 9
Current	36	1.2 ± 0.9	10	2.2 ± 2.7	22	3.8 ± 2.6	4	4.1 ± 3.2	36	12 ± 8	10	12 ± 10	36	12 ± 9	10	11 ± 6
Hydroxychloroquine																
Ever	27	1.1 ± 0.5	19	1.8 ± 2.2	15	2.9 ± 2.4	11	5.2 ± 2.5 ^†^	27	11 ± 7	19	13 ± 11	27	9 ± 7	19	15 ± 10
Current	16	1.4 ± 1.2	30	1.4 ± 1.7	10	3.6 ± 3.1	16	4.0 ± 2.4	16	13 ± 8	30	11 ± 9	16	12 ± 9	30	12 ± 9
Azathioprine																
Ever	25	1.2 ± 0.6	21	1.7 ± 2.1	16	3.7 ± 2.6	10	4.1 ± 2.8	25	12 ± 9	21	11 ± 8	25	12 ± 10	21	12 ± 7
Current	8	1.0 ± 0.5	38	1.5 ± 1.6	5	3.4 ± 2.7	21	4.0 ± 2.7	8	9.9 ± 5.6	38	12 ± 9	8	13 ± 12	38	11 ± 8
Cyclophosphamide (oral or IV)																
Ever	6	1.1 ± 0.5	40	1.5 ± 1.6	3	4.2 ± 4.2	23	3.8 ± 2.5	6	7 ± 5	40	13 ± 9	6	8 ± 10	40	12 ± 9
Current	4	2.1 ± 1.4	42	1.3 ± 1.5	2	N.A.	24	N.A.	4	13 ± 16	42	11 ± 8	4	13 ± 9	42	12 ± 9
Cyclosporin A																
Ever	9	1.5 ± 1.2	37	1.4 ± 1.6	5	4.3 ± 4.2	21	3.8 ± 2.5	9	11 ± 7	37	12 ± 9	9	11 ± 12	37	12 ± 8
Current	-	-	-	-	-	-	-	-	-	-	-	-	-	-	-	-
Mycophenolate mofetil																
Ever	8	1.3 ± 0.5	38	1.4 ± 1.6	5	2.9 ± 2.8	21	4.1 ± 2.6	8	15 ± 14	38	11 ± 7	8	11 ± 9	38	12 ± 9
Current	5	1.1 ± 0.5	41	1.5 ± 1.6	3	2.6 ± 3.4	23	4.0 ± 2.6	5	14 ± 10	41	12 ± 8	5	11 ± 9	41	12 ± 9

HPV, human papillomavirus; SLE, systemic lupus erythematosus; TLR, toll-like receptor; **^† ^***P <*0.05

Using regression analysis, HPV infection and a higher cumulative dose of prednisolone in the past 15 years were independent explanatory variables associated with down-regulation of TLR7 level in SLE (both *P *< 0.05) (Table 5). Moreover, HPV infection, a lower SLEDAI and a higher cumulative dose of hydroxychloroquine in the past 15 years were independent risk factors for down-regulating TLR9 level in SLE (all *P *< 0.05) (Table 5).

**Table 5:** Multiple linear regression with TLR7 and TLR9 expressions as independent variables in lupus patients

Risk factors	Adjusted coefficient (95%)	*P*-value	Adjusted R^2^
TLR7 level			0.592
HPV infection	-0.815 (-6.827 to -1.751)	0.002	
Cumulative dose of prednisolone in the past 15 years, gm	-0.356 (-0.014 to -0.001)	0.026	
High-risk HPV infection	0.217 (-1.487 to 3.926)	0.359	
Hydroxychloroquine therapy ever	0.012 (-1.919 to 2.046)	0.067	
			
TLR9 level			0.391
HPV infection	-0.313 (-10.395 to -1.170)	0.015	
SLEDAI	0.474 (0.841 to 2.576)	< 0.001	
Cumulative dose of hydroxychloroquine in the past 15 years, gm	-0.268 (-0.012 to 0.000)	0.041	
Duration receiving treatment of Azathioprine, month	-0.047 (-0.059 to 0.040)	0.706	

HPV, human papillomavirus; SLE, systemic lupus erythematosus; SLEDAI, systemic lupus erythematosus disease activity index; TLR, toll-like receptor

### TLRs and ISGs expressions are inhibited by oncogenic HPVs

**Figure 2:**
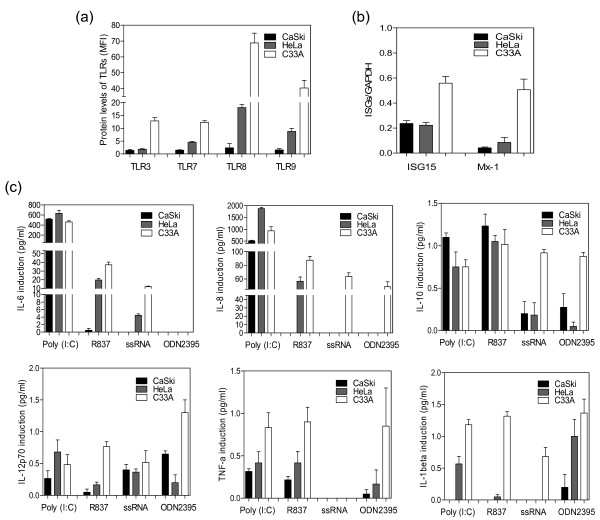
**Intracellular TLR-regulated pathways and ISGs are inhibited in oncogenic HPV cell lines**. **(a) **Flow cytometry of TLRs. Protein levels of TLRs 3, 7, 8, and 9 in Caski, HeLa or C33A cells were shown in bar charts as mean fluorescence intensity (MFI). **(b) **qRT-PCR of ISGs. Total RNA was extracted from Caski, HeLa or C33A cells. After reverse transcription, qRT-PCR was performed using ISG15 or Mx-1 primers. As positive control, GAPDH was amplified at exponential phase of 20 cycles. **(c) **Functionality of TLR-regulated pathways. CaSki, HeLa and C33A cells were stimulated for 24 h with ligands of TLRs 3, 7, 8, and 9 as indicated in the Figure. *Ex vivo *production of cytokines including interleukin (IL-6), IL-8, IL-10 and IL-12p70, tumor necrosis (TNF)-α and IL-1β in culture supernatant was measured by Cytometric Bead Array. Experiments were repeated three times in triplicate.

The protein levels of TLRs 3, 7, 8 and 9 were reduced in CaSki and HeLa when compared with C33A cells, respectively (Figure 2a). In addition, all expressions of intracellular TLRs were consistently reduced in CaSki compared to HeLa cells with the exception of TLR3 (Figure 2a). Similarly, ISG15 and Mx-1 gene expressions were down-regulated in CaSki and HeLa when compared to C33A cells, respectively (Figure 2b). Mx-1 expression was reduced in CaSki cells than HeLa cells. No significant differences of ISG15 gene level between oncogenic HPV types were observed (Figure 2b).

### Induction of inflammatory cytokines by TLR agonists was impaired in oncogenic HPVs

To investigate whether the reduced TLR protein levels has functional consequences for cytokine induction, HeLa, CaSki and C33A cell lines were incubated with TLRs 3, 7, 8 and 9 ligands, respectively. As shown in Figure 2c, the addition of TLR9 ligand ODN2395 led to a clear increase in IL-8, IL-10, IL-12p70, TNF-α and IL-1β in C33A cells, while a moderate increase was observed in the presence of the ligands for TLR7 (R837) and TLR8 (ssRNA). Conversely, the induction of all cytokines by TLR8 and TLR9 ligands were greatly impaired in oncogenic HPV types. Similarly, production of pro-inflammatory cytokines (IL-6, IL-8, IL-12p70, TNF-α and IL-1β) was strongly inhibited by TLR7 ligand in two cancer-derived HPV cell lines. Strikingly, no IL-6 and IL-8 inductions were observed in CaSki cells when stimulated with the ligand for TLR9, indicating that the oncogenic viral proteins are able to strongly inhibit the TLR9 pathways. In contrast to the inhibited TLRs 7, 8 and 9 pathways in oncogenic HPV types, TLR3 pathway was up-regulated in HeLa cells in comparison to CaSki and C33A cells and no obvious difference between the two different types was apparent (Figure 2c). The level of IFN-γ in three different cell lines was either undetectable or weak (data not shown).

## Discussion

Failure to induce an effective cellular immune response due to inefficient activation of innate immunity and ineffective priming of the adaptive immune response in lupus patients may lead to an increase in susceptibility and persistence of viral infection, including HPV. Emerging evidence has reported that nucleic acid-sensing TLRs seem to play a critical role in recognition of viral nucleic acids, both with regards to selectivity of their ligands, pathogens and differential recognition of foreign versus self nucleic acids [6,7]. An elevated level of these TLRs has been demonstrated in the PBMCs of lupus patients compared to healthy controls [11,12]. In contrast, this study showed that TLRs 7, 8 and 9 levels were significantly decreased in the cervical epithelial cells of HPV-negative lupus patients compare to healthy controls probably because of immunosuppressive therapy.

TLR antagonists, such as hydroxychloroquine, chloroquine and quinacrine, appear to be promising for a number of inflammatory and autoimmune diseases, especially in lupus patients [15,35]. Derivatives and small molecule analogues of chloroquine and quinacrine suppress the over-activation of immune responses [15,35]. These immunomodulatory drugs are antagonists of TLR9 and to a lesser extent, TLR7 and TLR8 [14,15]. Hydroxychloroquine, in particular, has been shown to interfere with the acidification of lysosomal compartments and subsequently inhibit intracellular signaling through TLR7 and TLR9 [36]. Glucocorticoids are widely used anti-inflammatory and immunosuppressive agents. Prednisolone could suppress the functions of TLR-stimulated human plasmacytoid dendritic cell [37]. Moreover, the administration of TLR7 agonist R848 induces a rapid local and systemic inflammatory response in mice that could be attenuated by glucocorticoids or TNF-α treatment [38]. Oral doses of prednisolone resulted in a marked reduction of TLRs 3, 4 and 7 activation by 70 to 90% compared with healthy controls, demonstrating the potent *in vivo *pharmacologic effect of this drug on TLR activity [39]. Although prednisolone and hydroxychloroquine has been widely used in SLE, *in vivo *effects of these agents on TLRs level in SLE have not been well elucidated. This study was the first to demonstrate a negative correlation between the cumulative dose of prednisolone and TLR7 level. Similarly, TLR9 level was found to be negatively correlated with the cumulative dose of hydroxychloroquine. These findings suggest that prednisolone and TLR antagonists may down-regulate the level of TLR7 and TLR9 in SLE, thereby decreasing the ability to clear HPV infection. Further prospective studies are warranted to confirm or refute this hypothesis.

Another novel finding from this study was the down-regulation of TLR3 and TLR7 levels in HPV-infected SLE compared to controls. Furthermore, significantly lower levels of TLR7 and TLR9 were observed in HPV-infected SLE than HPV-negative patients. A previous study reported that infection of human primary keratinocytes with HPV16 *E6 *and *E7 *recombinant retroviruses can inhibit TLR9 transcription and, hence, result in functional loss of TLR9-regulated pathways, suggesting that the modulation of TLR may be part of a mechanism by which HPV16 evades innate immunity [23]. In addition to down-regulating TLR9, we also reported a notable decrease in TLRs 7 and 8 in CaSki and HeLa compared to C33A cells. Our findings in cervical samples together with previous evidence [23], therefore, suggest that abolishing innate responses may be involved in viral persistence which may ultimately lead to carcinogenesis mediated by HPVs. Nonetheless, details of the pathogenic effect of oncogenic HPV on the host innate immune response remained to be elucidated.

The type I IFNs response is a strong and crucial moderator for the control of viral infections, in which the type I IFNs family members engage the ubiquitously expressed interferon-α/β receptor (IFNAR). Binding to IFNAR then stimulate more than 300 ISGs, which subsequently induce an antiviral state. The antiviral state is a collective term for limitation of viral replication, viral resistance of neighboring cells, and apoptosis of virally infected cells [16]. In this study, our data revealed that the antiviral ISG15 and Mx-1 gene expressions were inhibited by the two oncogenic HPV types, which is consistent with recent genomewide transcriptome studies of reduced ISGs expressions in high-risk HPV types [17-20], subsequently resulting in reduced levels of IFN and HPV DNA replication [17,20]. Functional data exploring whether the repression of theses intracellular TLRs and ISGs have functional consequences for inflammatory cytokines expression indicated that the induction of pro-inflammatory cytokines (IL-6, IL-8, IL-12p70, TNF-α and IL-1β) by TLR ligands (R837, ssRNA and ODN2395) were greatly impaired in two carcinogenic HPV cell types than HPV-negative cells. The down-regulation of the nucleic acid-sensing TLRs (TLRs 7, 8 and 9) ensures that the expression of inducible pro-inflammatory cytokines is minimized in order to prevent the expression of antiviral ISGs (ISG15 and Mx-1) of a biologically relevant antiviral response. Strikingly, no IL-6 and IL-8 inductions were observed in CaSki cells when stimulated with the ligand for TLR9, indicating that the oncogenic viral proteins are able to strongly inhibit the TLR9 pathway. Further studies are required to look into the details of the effect of oncogenic HPV on the avoidance of stimulation and down-regulation of the immune system, which permits persistence in the cervical epithelium and oncogenesis, is potential evidently part of an immune evasion strategy used by oncogenic HPV.

## Conclusion

In conclusion, prednisolone and TLR antagonist (hydroxychloroquine) may down-regulate expression levels of TLR7 and TLR9 in lupus patients, thereby decreasing the innate immune response to HPV infection. Furthermore, upon infection, HPV further down-regulate TLR7 and 9 levels which may favor viral persistence. Reduction of nucleic acid-sensing TLRs 7, 8 and 9 in carcinogenic HPVs ensures that the expression of inducible pro-inflammatory cytokines is minimized in order to suppress the expression of antiviral ISGs (ISG15 and Mx-1) of a biologically relevant antiviral response. Further studies to elucidate the role of immunosuppressants and HPV infectious on TLR expression in SLE, and whether topical TLR agonists may be useful as therapeutic tools for clearing oncogenic HPV infection in the cervical epithelial cells are warranted.

## Abbreviations

ASCUS: atypical squamous cells of undetermined significance; CBA: Cytometric Bead Array; CsA: cyclosporine A; DMEM: Dulbecco's modified Eagle's minimal essential medium/F12 medium; dsDNA: double-stranded DNA; dsRNA: double-stranded RNA; ELISA: enzyme-linked immunosorbent assay; FBS: fetal bovine serum; FC: flow cytometry; FITC: fluorescein iso-thiocyanate; FSC: forward scatter; HPV: human papillomavirus; IFN: interferon; IFNAR: interferon-α/β receptor; IL: interleukin; ISG: interferon-stimulated gene; MFI: mean fluorescence intensity; MMF: mycophenolate mofetil; Mx: myxovirus resistance; Pap: papanicolaou; PBMC: peripheral blood mononuclear cells; PBS: phosphate buffered saline; PCR: polymerase chain reaction; PE: phycoerythin; PRR: pattern-recognition receptor; RPMI: Roswell Park Memorial Institute medium; SIL: squamous intraepithelial lesion; SLE: systemic lupus erythematosus; SLEDAI: systemic lupus erythematosus disease activity index; SLICC: Systemic Lupus International Collaborating Clinics/American College of Rheumatology damage index; SSC: side scatter; ssRNA single-stranded RNA; TLR: toll-like receptor; TNF: tumor necrosis factor.

## Competing interests

The authors declare that they have no competing interests.

## Authors' contributions

PKSC, C-KW, C-CS, SCH, KS, MMYY, S-FY, T-HC, MCSW, EKL and L-ST contributed to the study design. S-LY, JLKC and ACMY contribute to the analysis and interpretation of data. S-LY, C-KW and L-ST contributed to manuscript preparation. S-LY contributed to statistical analysis. All authors read and approved the final manuscript.
